# Why Are Children Different in Their Daily Sedentariness? An Approach Based on the Mixed-Effects Location Scale Model

**DOI:** 10.1371/journal.pone.0132192

**Published:** 2015-07-31

**Authors:** Thayse Natacha Gomes, Donald Hedeker, Fernanda Karina dos Santos, Sara Pereira, Peter T. Katzmarzyk, José A. R. Maia

**Affiliations:** 1 Center of Research, Education, Innovation and Intervention in Sport (CIFI^2^D), Kinanthropometry Lab, Faculty of Sport, University of Porto, Porto, Portugal; 2 Department of Public Health Sciences, University of Chicago, Chicago, Illinois, United States of America; 3 Department of Physical Education and Sports Science, CAV, Federal University of Pernambuco, Vitória de Santo Antão, Pernambuco, Brazil; 4 Pennington Biomedical Research Center, Louisiana State University, Baton Rouge, Louisiana, United States of America; Leibniz Institute for Prevention Research and Epidemiology (BIPS), GERMANY

## Abstract

This study aimed to investigate the between- and within-individual variability in sedentary time over seven days, using a mixed-effects location scale model. The sample comprised 686 Portuguese children (381 girls) aged 9–11 years, from 23 schools. Sedentary time was estimated by the Actigraph GT3X+ accelerometer, which was used 24 hours/day for 7 consecutive days; height, sitting height, and weight were measured, BMI was computed (WHO cut-points were used to classify subjects as normal weight or overweight/obese), and maturity offset was estimated. Information regarding the home environment was obtained by questionnaire. Results revealed that (i) children were more sedentary on Friday, but less so on Saturday and Sunday (compared to Monday), with significant variation between- and within-subjects (between-subject variance=0.800, within-subject variance=1.793, intra-subject correlation=0.308); (ii) there is a sex effect on sedentariness, with boys being less sedentary than girls (p<0.001), and the between-subject variance was 1.48 times larger for boys than girls; (iii) in terms of the within-subject variance, or erraticism, Tuesday, Wednesday and Friday have similar erraticism levels as Monday (Thursday has less, while Saturday and Sunday have more); in addition, girls (variance ratio=0.632, p<0.001), overweight/obese children (variance ratio=0.861, p=0.019), and those later mature (variance ratio=0.849, p=0.013) have less erraticism than their counterparts; (iv) the within-subject variance varied significantly across subjects (scale std dev=0.342±0.037, p<0.001); and (v) in the fixed part of the model, only biological maturation was positively related to sedentariness. This study demonstrated that there is significant between- and within-subject variability in sedentariness across a whole week. This implies that a focus on intra-individual variability, instead of only on mean values, would provide relevant information towards a more complete map of children’s sedentary behaviour, which can be helpful when developing more efficient strategies to reduce sedentariness.

## Introduction

The last years witnessed an augmented interest in monitoring and understanding sedentary behaviours [1], their correlates [2], and their relationships with health hazards and reduced quality of life [3,4]. There is now compelling evidence that children and adolescents spend a large proportion of their day in sedentary behaviours [5]. However, sedentary behaviour differs among youth according to their intrapersonal traits and interpersonal characteristics, as well as built and physical environmental factors [5,6,7]. For example, several studies have identified distinct clusters of youth based on their levels and patterns of sedentary behaviour alongside their physical activity levels [1,8,9]. Further, sex [10], age [11], and maturity status [12] have also been identified as correlates of sedentariness.

Notwithstanding the intensified interest in having a more comprehensive understanding of patterns and correlates of sedentary behaviour [5,10,13,14], the available research has focused largely on mean differences [15], used sets of covariates in multiple regression models to predict sedentariness [2], or studied contextual inter-individual differences using multilevel models [16,17,18]. To our knowledge, exploring factors related to intra-individual variability in sedentary behaviour across days, which can help to better understand observed between-subject differences and effectiveness of interventions beyond mean changes, has never been addressed.

Studying variability in intra-individual differences in daily behaviours is relevant to provide an understanding of patterns of sedentary behaviour over time [19,20]. Further, seven-day objective monitoring is an acceptable window to study different physical activity and sedentary behaviour expressions [21]. Thus, the purpose of the current study is to investigate the between- and within-individual variances in sedentariness over seven days of objective monitoring, in order to answer the following questions: (i) Is there a trend in children’s sedentary behaviour over an entire week? (ii) Is this trend similar in boys and girls? (iii) Is there appreciable variability in sedentary time across days? (iv) Does variability in sedentary behaviour differ for each subject or is its magnitude similar for all children? (v) Which variables are associated with variability in sedentary behaviour? To answer these questions, we used a mixed-effects location scale model [22,23] which allows both the mean and variance structures to be modelled in terms of covariates.

## Methods

### Sample

The sample of this study is part of the International Study of Childhood Obesity, Lifestyle and the Environment (ISCOLE), a research project conducted in 12 countries from all major world regions. In short, ISCOLE aims to determine the relationship between lifestyle behaviours and obesity in a multi-national study of children, and to investigate the influence of higher-order characteristics such as behavioural settings, and the physical, social and policy environments, on the observed relationship within and between countries [24]. Since the purpose of ISCOLE was to study children with a mean age of 10 years, ranging from 9 to 11 years, our sample recruitment was only done in 5^th^ grade students. A total of 777 5^th^ grade Portuguese children (419 girls), aged 9–11 years, were assessed, and after the inclusion criteria (accelerometer valid data for at least 4 days, as described below), the final sample comprised 686 children (381 girls). These students belong to 23 schools from the metropolitan area of Porto, North of Portugal, which were selected from a list provided by the North Regional Education Directory Board, taking account their location (schools should be located in different socio-economic neighbourhoods).

After a first initial contact with a physical education teacher from each school, the project was presented to the physical education department. Following their approval, the project was then presented to the school principal as well as to the parental council; it was only after obtaining these approvals that the project was implemented in each school. All 5^th^ grade children were invited to be part of ISCOLE; however, only children aged between 9 and 11 years were classified as “eligible” to be part at the project. From those “eligible” children, a sample of ≈30–40 children per school was randomly selected (50% for each sex). Non-response was negligible (response rate was 95.7%).

Data were collected from September 2011 to January 2013. All assessments were done during a full week per school by trained personnel from the Kinanthropometry Laboratory of the Faculty of Sport (University of Porto) following certification from the ISCOLE Coordinating Center; the questionnaires were answered by each child, at their school, after anthropometric measures were taken, and under the supervision of at least one ISCOLE staff member. The study protocol was approved by the University of Porto ethics committee, as well as by the schools’ directorate councils. Written informed consent was obtained from parents or legal guardians of all children. All data collection and management activities were performed and monitored under rigorous quality control procedures, implemented by the ISCOLE Coordinating Center, as previously described in detail [24].

### Anthropometry

Height, sitting height, and weight measures were taken according to standardized ISCOLE procedures [24]. For height and sitting height, children were measured without shoes, with head positioned in the Frankfurt Plane, using a portable stadiometer (Seca 213, Hamburg, Germany); height was measured with children fully erect, feet together, and at the end of a deep inhalation, while sitting height was measured with children seated on a table with legs hanging freely and arms resting on the things. Leg length was computed by subtracting sitting height from standing height. Weight was determined using a portable Tanita SC-240 body composition analyzer (Arlington Heights, IL), with children wearing light clothes and without shoes or socks. Each child was measured twice and, when necessary, a third measurement was taken if the difference between the previous two was outside the permissible range for each measure and its replica (0.5 cm for height and sitting height; 0.5 kg for weight). The mean value of each measured variable was used for analysis.

Body mass index (BMI) was calculated using the standard formula [weight(kg)/height(m)^2^], and subjects were classified into two groups (normal weight and overweight/obese) according to the cut-off points from the World Health Organization (WHO), based on BMI z-scores (normal-weight: <+1SD; overweight/obese; ≥+1SD) [25].

### Family data

Family information was obtained by a questionnaire completed by parents or legal guardians [see ISCOLE Demographic and Family Health Questionnaire [24]]. The questionnaire collected information on basic demographics, ethnicity, family health and socioeconomic factors, and was answered by parents/legal guardians during the same week their children were assessed at school. For the present study, we used information about media availability in the child’s bedroom. Media availability in the child’s bedroom was determined by asking parents if children had a computer or video game in their bedroom. The existence of TV in the child’s bedroom was informed by the children. Using information regarding media availability in the child’s bedroom (TV, computer or game), a “media bedroom” variable was computed to determine if there is, at least, one media available at children’s bedroom; so subjects were classified as “having media in bedroom” or “not having media in the bedroom”.

### Sedentary Time and Sedentary Behaviour

Actigraph GT3X+ accelerometers (ActiGraph, Pensacola, FL) were used to monitor sedentary time. Children wore the accelerometer at their waist on an elasticized belt, placed on the right mid-axillary line 24 hours/day, for at least 7 days, including 2 weekend days. To be eligible for this analysis, children had at least 4 days (from which at least one of them should be a weekend day) with a minimum of 10 hours of wear time per day; 686 children fulfilled this condition. Accelerometer information was divided into daytime activities and nocturnal sleep time using an automated algorithm, and any sequence of at least 20 consecutives minutes of zero activity counts during awake period was considered as non-wear time [26,27]

Sedentariness is a multi-faceted characteristic that includes behaviour at work/school, at home, during transport, and in leisure-time including screen-time, motorized transportation, and sitting (to read, talk, do homework, or listen to music) [28]. In the present study, sedentary time objectively measured by the accelerometer is our primary dependent variable, and is defined as equal to or less than 25 counts/15 seconds as advocated by Evenson et al. [29]. Further, information was also collected about children’s sedentary behaviour, by asking them about time spent watching TV during school days [ISCOLE Diet and Lifestyle Questionnaire [24]], and they were classified as ≤2hours/day or >2hours/day.

### Biological maturation

Using information on sex, age, and physical growth characteristics (sitting height, leg length, stature and body mass), an estimate of biological maturity, namely somatic maturation, was obtained using the Mirwald et al. [30] maturity offset method. This method estimates, in decimal years, the status of the child relative to their age at peak height velocity (PHV) occurrence. A positive maturity offset expresses the number of years a child is beyond PHV; a negative maturity offset indicates the number of years before PHV.

### Data Analysis

All exploratory data analysis and descriptive statistics, as well t-tests, were done in SPSS 20, and MS Excel was used to plot differences in sedentariness trajectories and patterns of two boys and girls with similar mean sedentariness values. The specifics of the mixed-effects location scale model has been described elsewhere in great detail [22,23]. Briefly, the model for the sedentary measurement y, of child i (i = 1, 2, 3, …, N subjects) on day j (j = 1, 2, 3, …, n_i_ days) is,

yij = x´ijβ + υi + εij, (1)

where x_ij_ is the vector of regressors and **β** is the corresponding vector of regression coefficients. The regressors can either be at the subject level, vary across occasions, or be interactions of the subject-level and occasion-level variables. The random subject effect υ_i_ indicates the influence of subject i on his/her sedentariness measures; these random effects are assumed to be normally distributed with zero mean and variance $\sigma_{v}^{2}$. The errors ε_ij_ are also assumed to be normally distributed with zero mean and variance $\sigma_{\varepsilon }^{2}$, and independent of the random effects. Here $\sigma_{v}^{2}$ represents the between-subjects (BS) variance and $\sigma_{\varepsilon }^{2}$ is the within-subjects (WS) variance. The mixed-effects location scale model allows both of these variances to be modelled in terms of regressors using log link functions (to ensure positive variances). The coefficients from these variance models can be exponentiated to yield variance ratio estimates for the regressors (i.e., relative change in the variance per unit change in the regressor). Additionally, a random subject effect is included in the WS variance specification, which permits this variance to vary at the subject level, above and beyond the influence of regressors [22,23].

## Results

Table 1 shows descriptive statistics (Mean±SD and percentages) for the sample. There is a relatively high frequency (about 46%) of overweight/obesity. On average, children are about 2 years from their PHV. Almost 90% of the children reported 2 or less hours watching TV on week days, and 80% of them have at least one electronic media (TV/PC/game) in their bedroom.

**Table 1:** Descriptive characteristics of children.

VARIABLE		Mean±sd or Percentage (%)
BMI (kg·m^-2^)		19.5±3.4
Maturity Offset (years to PHV)		-1.90±0.89
BMI (classification)		
	Normal weight (0)	54.2%
	Overweight/Obese (1)	45.8%
Time watching TV on school days		
	≤2 hours/day (0)	89.7%
	>2 hours/day (1)	10.3%
Electronic media in bedroom		
	No (0)	19.8%
	Yes (1)	80.2%

**Fig 1:**
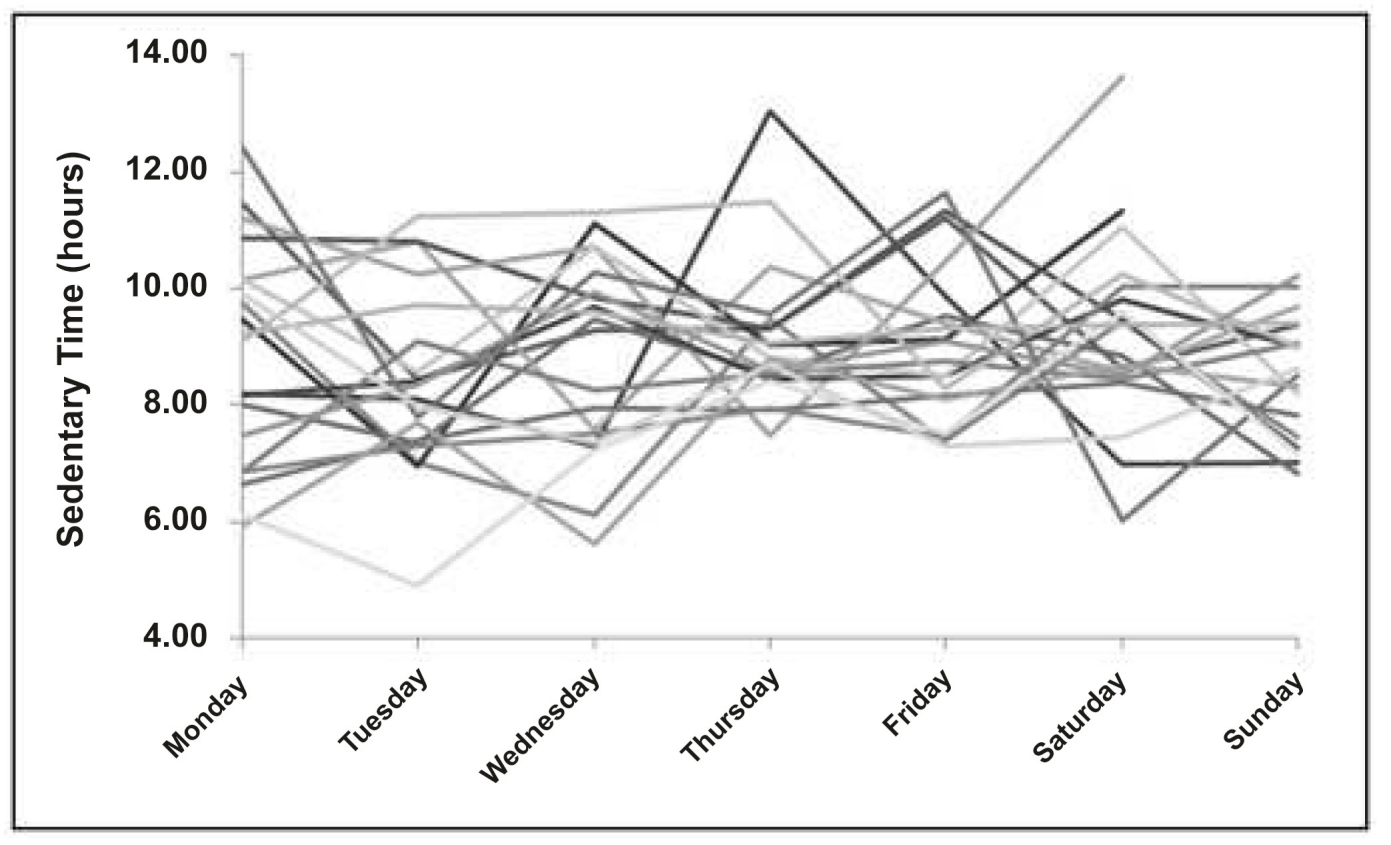
Time spent in sedentary behaviour over a week, for boys.

Time spent in sedentary behaviour for each of the 686 subjects over an entire week is presented in Table 2 (for both sexes), and Figs 1 (for boys) and 2 (for girls) show time spend in sedentariness from 20 random boys and girls. On average, boys spend less time in sedentary behaviour than girls in all week days (Monday to Friday), but no statistically significant difference was observed on Saturday and Sunday. The data reveal variability in sedentary time, with differences in their trajectories from Monday to Sunday. To give a sense of this variability at the subject level, we plot the gender-specific means in Fig 3, along with one subject from each group that is highly variable, and one subject that is rather consistent. Additionally, the two subjects in each gender group have the same average across time, though they differ quite a lot in terms of their variability. Individual difference in the within-subject variation is precisely what the mixed-effects location scale model allows for and attempts to explain.

**Table 2:** Mean±standard deviation for daily sedentary time (hours·day^-1^) for boys and girls.

Days	Girls	Boys
Monday*	9.52±1.46	8.92±1.66
Tuesday*	9.62±1.44	8.95±1.60
Wednesday*	9.55±1.40	8.88±1.65
Thursday*	9.43±1.44	8.87±1.64
Friday*	9.55±1.57	9.23±1.61
Saturday	9.08±1.60	8.86±1.83
Sunday	8.96±1.59	9.04±1.88

*statistically significant difference between boys and girls (p<0.05)

**Fig 2:**
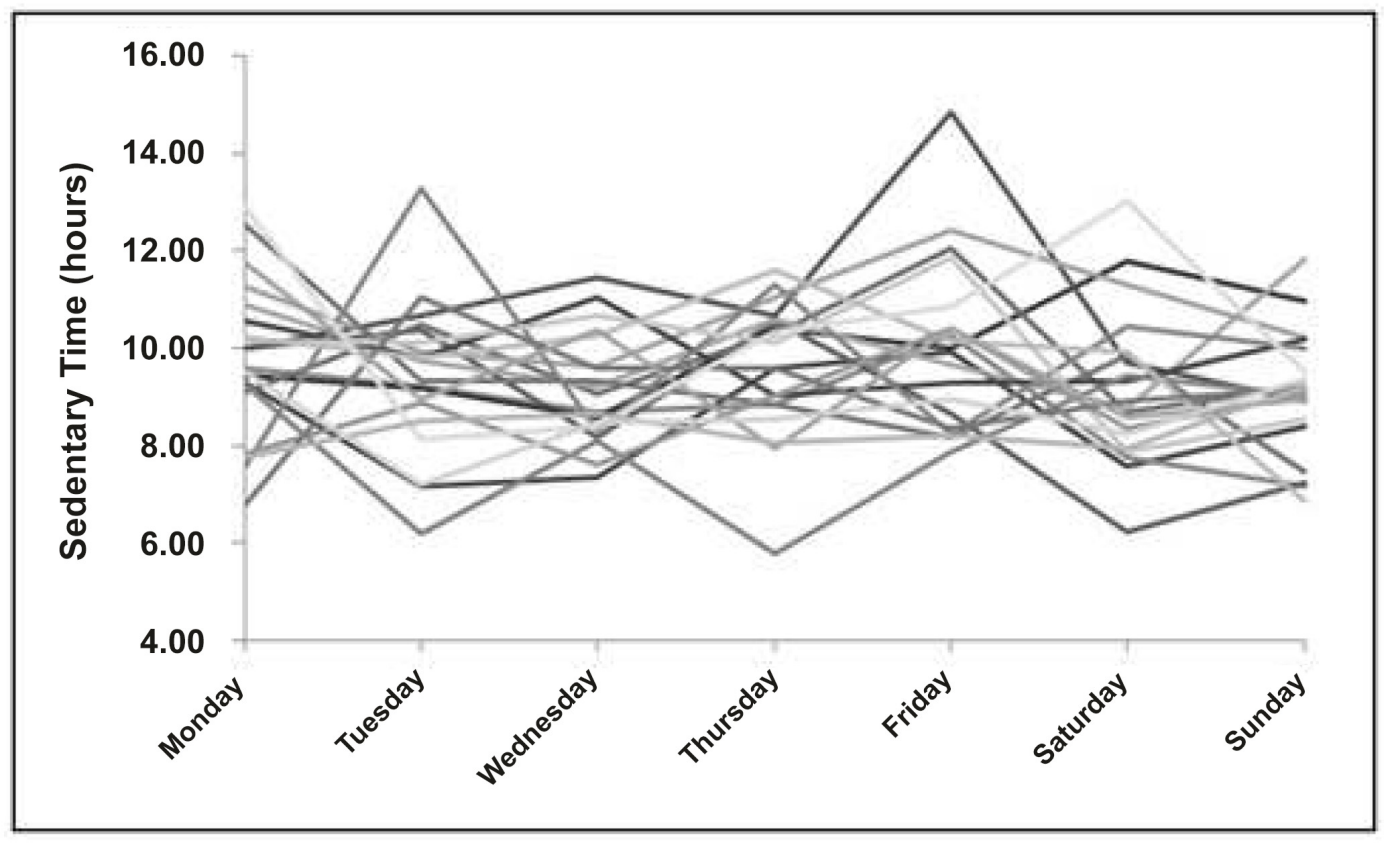
Time spent in sedentary behaviour over a week, for girls.

**Fig 3:**
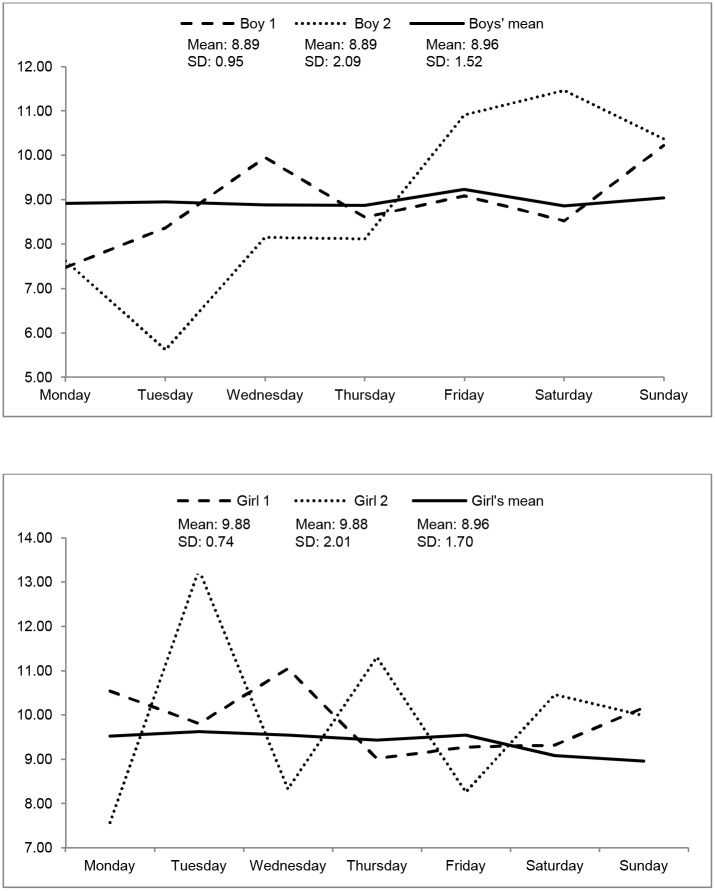
WS differences in sedentariness along a whole week, for 2 boys (up) and two girls (down), with same mean sedentariness time across the week.

Results concerning the five research questions are included in Table 3. Note that although we have a three-level model, daily observations nested within children nested within schools, the software MIXREGLS does not accommodate 3 levels. In any case, we add school-indicators (22 dummy variables given that we have 23 schools) into the model. The results did not change in terms of the overall interpretation, and so, for simplicity, we are presenting the results from the models without these indicators.

**Table 3:** Parameter estimates (±standard errors) of the four models.

Model parts	Model 1	Model 2	Model 3	Model 4
	β±SE	β±SE	β±SE	β±SE
**Fixed part**				
Intercept	9.243±0.062**	9.433±0.068**	9.451±0.066**	9.740±0.168**
Tuesday	0.070±0.074^ns^	0.069±0.074^ns^	0.077±0.067^ns^	0.073±0.067^ns^
Wednesday	0.004±0.074^ns^	0.005±0.074^ns^	0.008±0.068^ns^	0.006±0.068^ns^
Thursday	-0.064±0.073^ns^	-0.064±0.073^ns^	-0.058±0.065^ns^	-0.064±0.065^ns^
Friday	0.167±0.074**	0.167±0.074***	0.184±0.071**	0.187±0.071**
Saturday	-0.261±0.073**	-0.260±0.073**	-0.265±0.076**	-0.267±0.076**
Sunday	-0.267±0.074**	-0.267±0.074**	-0.294±0.077**	-0.297±0.076**
Sex		-0.430±0.079**	-0.475±0.079**	-0.104±0.165^ns^
BMI				-0.151±0.090*
Maturity Off				0.242±0.090**
Time TV				0.183±0.126^ns^
Media				0.072±0.095^ns^
**Between Subject (BS) variance**				
Intercept	-0.232±0.073**	-0.482±0.104**	-0.401±0.098**	-0.436±0.099**
Sex		0.390±0.147**	0.299±0.145**	0.330±0.146**
**Within Subject (WS) variance**				
Intercept	0.584±0.022**	0.584±0.022**	0.330±0.071**	0.581±0.132**
Tuesday			-0.106±0.094^ns^	-0.109±0.094^ns^
Wednesday			-0.023±0.092^ns^	-0.024±0.092^ns^
Thursday			-0.199±0.093**	-0.204±0.093**
Friday			0.124±0.092^ns^	0.130±0.092^ns^
Saturday			0.369±0.091**	0.370±0.091**
Sunday			0.360±0.092**	0.356±0.092**
Sex			0.199±0.055**	0.459±0.115**
BMI				-0.150±0.064**
Maturity Off				0.164±0.066**
Time TV				-0.072±0.089^ns^
Media				0.026±0.068^ns^
**Random location (mean) effect on WS variance**				
Loc eff			0.086±0.031**	0.080±0.031**
**Random scale standard deviation**				
Std dev			0.348±0.037**	0.342±0.037**
**Deviance (-2 Log L)**	16892.843	16855.689	16712.490	16694.754

^ns^ = non-significant;

*<0.10;

**<0.05;

-2 Log L = -2 Log-likelihood

The models are presented in increasing complexity, starting with a simple multilevel model to assess levels of BS and WS heterogeneity. Thus, it was started with a conventional random intercept model that only includes covariate effects on the mean (model 1). Then, in model 2, gender was added as a covariate for both the mean and BS variance model. Models 3 and 4 then introduced the novel aspects, which is the WS variance modelling in terms of covariates and (random) subject effects. The difference between models 3 and 4 is the inclusion of the variables listed in Table 1 in both the mean and WS variance modelling. So, model 1 addressed the question of a possible trend in sedentary behaviour across days of the week (Monday is the reference day). On average, the number of sedentary hours on Monday was 9.2±0.06, and no significant differences were found for Tuesday, Wednesday and Thursday (relative to Monday). Children were more sedentary on Friday, but less so on Saturday and Sunday (parameter estimates have a negative sign). There is significant variation BS across days as these estimates are from log link functions, the BS variance estimate is exp(-0.232) = 0.79, and the WS estimate is exp(0.584) = 1.79, resulting in a intra-subject correlation of 0.306. Concerning question two (model 2), boys and girls (reference category) differ in the average level of sedentariness, with boys being less sedentary than girls, and the BS variance was seen to be 1.48 times larger for boys than girls (exp(0.390) = 1.48). Taken together, boys are, on average, less sedentary, but more heterogeneous than girls.

Model 3 addressed questions three and four, and fits significantly better than model 2 (likelihood-ratio chi-square = 16855.689–16712.490 = 143.2, which on 9 degrees of freedom is highly significant, p<0.0001). The fixed and BS variance parts are similar to model 2. The novelties are located first in the WS variance modelling, and second in the random scale standard deviation. Tuesday, Wednesday and Friday have similar variability to Monday, i.e., same erraticism; Thursday has less, while Saturday and Sunday have more; further, significant sex differences were observed in this erraticism. The random scale standard deviation indicates whether the WS variance varies across subjects (over and above the effects of regressors on the WS variance). In other words, do subjects differ in how consistent/erratic they are in sedentary behaviour? It is highly significant, and so the degree of sedentary behaviour consistency/erraticism does vary significantly across subjects (over and above the day of week and sex effects).

Model 4 addressed question five about which variables, from the previously described set (Table 1), might be associated with this variability. Results in the fixed part of the model showed that sex, media, and time watching TV do not affect mean sedentariness time; BMI category is marginally associated; whereas children advanced in their biological maturation are more sedentary. There are significant inter-individual (BS) differences among subjects, as well as a significant sex effect. Erraticism is now significant only on Thursday, Saturday and Sunday; further, from the set of covariates, sex, BMI category, and maturation were significant, meaning that girls (variance ratio = 0.632), overweight/obese children (variance ratio = 0.861), and those less mature (variance ratio = 0.849) have less erraticism than their counterparts. In terms of the effect of a person’s mean on their WS variance, note that those subjects above average exhibited more erraticism than their peers with lower averages; in addition, the random scale standard deviation remains highly significant confirming that the WS variance does vary across subjects.

## Discussion

This study aimed to investigate the influence of several predictors of the between- and within-subject differences and variability in their sedentariness across seven days, following a set of five different questions. The first question was if there was a trend in children’s sedentary behaviour over an entire week. Results showed that children tended to be more sedentary on Friday and less so on the weekend, when compared to Monday. Previous studies [31,32,33], aiming to identify children differences between weekdays and weekend days in their sedentary time, showed that they are more sedentary during weekdays. For example, Carson et al. [31] investigated the levels and bouts of measured sedentary time accumulated during different days of the week by 12–15 years old Australian girls, and reported that their sedentary time was higher on weekdays as compared to weekend days. Similarly, Steele et al. [32] also examined volume and patterns of sedentary activities during different segments of the week in UK children aged 9–10 years, and Harrington et al. [33] found higher levels of sedentary behaviour during weekdays when compared to weekend days among Irish female adolescents. This consistent pattern in sedentariness during the week days of children from different countries is governed, to some extent, by their school schedules and activities, which contribute to more sedentary behaviour among students. Harrington et al. [33] highlighted that during the period children spend at school they usually accumulate more sedentary time, implying that the school setting appears to impose sedentariness in children, especially promoting unbroken continuous periods of sitting. On the other hand, during the weekend, children have more opportunities to be physically active, spending less time in sedentary activities, such as sitting, reading or using the computer.

The second question addressed the issue of sex differences in sedentary behaviour, and boys were not only less sedentary but also showed more heterogeneity in their sedentariness across the seven days. Sex differences on average levels of both physical activity and sedentariness have been previously reported showing that girls usually tend to be more sedentary [10,34], which can be related to their options for sedentary activities during their leisure time (such as reading, listening to music, socializing with friends), while boys tend to engage in more physically activities (such as sport participation, competitive games) [35].

Since sex differences were found in the children’s sedentary behaviour heterogeneity (model 2, α = 0.390±0.147, p = 0.008), this was further explored across all days as well as its difference between boys and girls (model 3) using the novelty and flexibility of the mixed-effects location scale model [22,23] which allows the WS variance to be modelled in terms of regressors and also allows subjects to vary in their consistency/erraticism. Results showed significant WS variance across days, meaning that children do not have the same sedentary pattern across all days. To our knowledge this is the first time that the idea of BS and WS variance in sedentariness has been jointly explored. It is expected that children vary in their physical activity and sedentariness between and within days [36], and some studies have investigated the within-day variability in sedentariness in children and adolescents considering the school-time and outside school-time. For example, Harrington et al. [33] did not find differences in girls’ sedentary time between these two periods of the day, but reported that these adolescents tend to accumulate more sedentary bouts during school time. On the other hand, Steele et al. [32] reported period and sex differences in sedentary time during the day, with boys spending more time in sedentary activities out of school while no differences in sedentariness between “in-school” and “out-of-school” periods were observed in girls. However, these “trends” may not be similar in all children given that the analysis by Harrington et al. [33] and Steele et al. [32] were based on averages and/or percentages of the total time. We think that differences in children’s sedentariness may be more properly addressed by the modelling of the WS variance, which is not always taken into account. Previous research specifically addressing the issue of intra-individual differences, although in aging, reported by Hertzog and Nesselroade [37], clearly stated that using averages to describe changes is not always the best way to detect key features of developmental changes and/or short-term differences. Further, they showed that change can vary within a person over weeks, even when the time of the day and day of the week of testing is kept constant. Additionally, Epstein [38] pointed out that not everyone is equally predictable, highlighting that a significant WS variance exists and that it should be taken into account, independent of the outcome variable. Fig 3 highlights the WS differences in sedentariness along a whole week in two boys and two girls. Children with the same (or similar) mean sedentariness time show different sedentariness trajectories and patterns during the week, revealing that WS variance exists and that should be taken into account when studying correlates of sedentary behaviour.

The usual approach in population studies, taking into account only the mean values of the entire sample, is based on the assumption that results and the distribution of the variables at the population level somehow reflect within-person processes, which allow the generalization from the population to the individual [39]. However, as we showed in Fig 3, results at the person level are different from those at the sample level, and as reported by Hamaker [39], when there are individual differences that cannot be ignored, it is necessary to “investigate to see what predicts them or what they predict” ([39], p. 52). This was the main drive of our last question (results in model 4) addressing the simultaneous effects of sex, BMI categories, biological maturation, time watching TV, and electronic media in the bedroom in children mean differences (expressed in the fixed part of the model) and intra-individual variability (expressed in the WS variance) in sedentariness across the seven days. From the set of covariates tested, only biological maturation was significantly related to mean levels of sedentariness, indicating that more mature children tend to be more sedentary than their less mature peers. This result is in accordance with previous studies where this association was reported [12,40]. For example, Brodersen et al. [12] described that more advanced puberty was associated with greater sedentariness in youth. Similarly, Machado Rodrigues et al. [40] found that maturity status is a significant predictor of sedentariness, but only in boys. Since girls mature earlier than boys, it is possible that differences in maturation timing and tempo may also explain sex differences in sedentariness, as described in associations with physical activity and exercise [41,42,43], but this issue is still unclear [40].

From those variables related to the WS variance in sedentariness, sex (τ = 0.459±0.115, p<0.001), BMI categories (τ = -0.150±0.064, p = 0.019) and biological maturation (τ = 0.164±0.066, p = 0.013) showed significant effects on the variance in sedentariness, meaning that girls, overweight/obese children and those late in their maturation have a lower erraticism in their sedentariness. Further, this result reinforces the need for a careful study of WS variance. Since, in general, the covariates at WS level differ from those at BS level as well as from those at mean level of sedentariness, generalization of the results about covariates from the inter-individual level can extend to intra-individual variability, or considering erraticism a nuisance, may not be appropriate. In addition, as highlighted by Molenaar (cited by Hamaker [39]), this generalization from the population to the individual is only appropriate when the population moments (means, variances, and covariances) are identical to the corresponding within-person moments, which are not the case in almost situations.

Some limitations in the present study should be discussed. Firstly, since children spend a substantial part of their awake time at school, the school environment can have a relevant role at WS variance in sedentariness; however, school context characteristics were not included in the model, and its effect on children’s sedentariness or physical activity is not always clear, given the large variability found in the school effects’ intraclass correlation going from ≈0.06 to ≈0.36 [18,44]. Secondly, it is possible that children vary in their sedentariness also within a day, due to their different surroundings (e.g., school, home, sports club), and studies of sedentariness variance during the day can offer relevant information about children’s patterns of sedentary behaviour. The use of ecological momentary assessment approaches may be highly useful in unravelling this issue [45,46]. Thirdly, the sample comes from only one Portuguese region, meaning that results cannot be generalized to other Portuguese children. However, in data not shown, similar results were observed in some sample characteristics between our sample and others from previous studies, namely in the prevalence of overweight/obesity [47] and socioeconomic status distribution [48]. In spite of these limitations, several strengths should be pointed out: (1) to our knowledge, this is the first study that explored WS variance in sedentariness, highlighting the relevance of understanding the BS variance, the WS variance as well as their possible predictors; (2) the use of an objective method to estimate sedentariness during a whole week; (3) the use of standard methods and reliable data; (4) and the use of the mixed-effects location scale model to study the complexity of BS and WS variance in sedentariness.

## Conclusions

This study showed that children are significantly different in their sedentariness during the days of the week, and tend to be less sedentary during the weekend, (suggesting that the school context may play a relevant role), and that sex difference exists regarding to sedentariness. Within-child consistency/erraticism showed high variability across days, meaning that children do not have the same sedentariness patterns along the week; further, sex, BMI, and biological maturation have significant effects on the sedentariness variance. Since results from between- and within-child are not the same, namely in their correlates, this reinforces the need to a deeper investigation on intra-individual variability above and beyond the normative view of mean values and heterogeneity among subjects. In addition, results found at the inter-individual level do not generalize to intra-individual level. The approach used in the mixed-effects location scale model showed to be very important in providing detailed information for a better understanding of correlates that best explain intra-individual sedentariness consistency/erraticism. Taken together, these findings provide evidence that a more complete map of children’s patterns in sedentary behaviour will be highly important when designing intervention strategies to reduce their sedentariness and associated health hazards, and that within-child variance should not be neglected.
